# Rapsyn overexpression improves neuromuscular junction and skeletal muscle function after tourniquet-induced mouse hindlimb ischemia-reperfusion injuries

**DOI:** 10.3389/fbioe.2026.1906715

**Published:** 2026-10-15

**Authors:** Huiyin Tu, Emmanuel Adu-Agyekum, Aaron N. Barksdale, Michael C. Wadman, Yu-Long Li

**Affiliations:** Department of Emergency Medicine, University of Nebraska Medical Center, Omaha, NE, United States

**Keywords:** ischemia/reperfusion, neuromuscular junction, rapsyn, skeletal muscle, tourniquet

## Abstract

Tourniquet-induced hindlimb ischemia-reperfusion (tourniquet/IR) results in the fragmentation of nicotinic acetylcholine receptors (nAChR) clusters in the neuromuscular junction (NMJ) and impairs neurally controlled skeletal muscle contraction. Rapsyn is a cytoplasmic nAChR-associated protein and participates in the nAChR assembly at the NMJ. Here, we observed whether the down-expression of rapsyn mediated the structural damage and functional impairment of the NMJ and skeletal muscle from tourniquet/IR injuries in mouse hindlimb. Unilateral hindlimb of C57BL/6 mice was subjected to 3 h of ischemia by placing a rubber band and followed by 6 weeks of reperfusion (tourniquet/IR). Ad-m-RAPSN was injected into the gastrocnemius muscle to increase rapsyn levels. LV-m-Rapsyn shRNA was used to knockdown rapsyn levels. After 6 weeks of sham treatment or tourniquet/IR, skeletal muscle contraction, endplate potentials (EPPs), and morphology of NMJs were assessed. Compared to sham mice, NMJs in the gastrocnemius muscle were significantly fragmented with low rapsyn expression, and the amplitude of EPPs and muscle contraction were also decreased in tourniquet/IR mice. Ad-m-RAPSN transfection into tourniquet mice increased rapsyn expression, reduced fragmented NMJs (38.2% ± 4.0%, compared to 74.7% in tourniquet/IR alone, P < 0.05), and raised EPP amplitudes and skeletal muscle contraction. Conversely, rapsyn shRNA-downregulated rapsyn expression in normal mice resulted in the NMJ fragmentation and neuromuscular dysfunction. These data suggest that tourniquet/IR decreases rapsyn expression in the skeletal muscle, which results in the nAChR cluster fragmentation and impairs skeletal muscle contractile function. Rapsyn overexpression could be a therapeutic strategy to improve the morphological and functional recovery of the NMJ as well as skeletal muscle contraction from tourniquet/IR injuries.

## Introduction

Skeletal muscles are found throughout the body, and contractions of skeletal muscles provide stability and power for all body movements. Skeletal muscles require motor nerve innervation to produce muscle contraction or maintain muscle tone for the avoidance of muscle atrophy. The neuromuscular junction (NMJ) is a specialized synapse that connects motor neurons and skeletal muscle fibers (Li et al., 2018). In vertebrates, the muscle fiber contains a specialized membrane region known as the “motor end plate,” where nicotinic acetylcholine receptors (nAChRs) are densely packed (Sanes and Lichtman, 2001; Sanes and Lichtman, 1999). Acetylcholine (Ach), a neurotransmitter released from motor neurons, binds to the nAChRs on the cell membrane of the skeletal muscle fibers to generate a local depolarization known as the endplate potential (EPP) (Ruff, 2011).

Normally, efficient neuromuscular transmission is highly reliable and a single EPP is sufficient to reach the threshold for the generation of an action potential that eventually results in muscle contraction (Wood and Slater, 2001). In the NMJ, the high density of nAChRs is clustered in the postsynaptic membrane as safety mechanism in that the excess depolarization of the postsynaptic membrane in response to each nerve impulse ensures the occurrence of skeletal muscle contraction (Ruff, 2011; Wood and Slater, 2001). There are several key skeletal muscle-associated NMJ-organizing proteins for nAChR cluster formation, such as agrin, LrP4, MuSK, DoK7, Dok7, and rapsyn (Li et al., 2018). Of those, as a scaffolding protein, rapsyn binds with nAChRs to aggregate them at high density into clusters (Xing et al., 2020), which are essential for efficient neuromuscular transmission. Rapsyn does not regulate the nAChR expression (Frail et al., 1989), but it can contribute to the nAChR stabilization on the postsynaptic membrane (Han et al., 2000).

Tourniquet is commonly used for severe limb hemorrhage and is often necessary to save life (Ko et al., 2024). However, clinical studies have shown that tourniquet application and subsequent release (tourniquet/IR) destroy NMJ structure and impair NMJ and skeletal muscle function, even cause complete limb paralysis and need secondary amputation (Aho et al., 1983; Appell et al., 1993; Clasper et al., 2009; Daya et al., 2008; Fludger and Bell, 2009; Gillani et al., 2012; Kam et al., 2001; Malo et al., 2015; Noordin et al., 2009; Rorabeck and Kennedy, 1980; Shlaifer et al., 2017). These tourniquet/IR-induced complications have led to the deemphasis, and limitations of clinical tourniquet use (Clasper et al., 2009; Lu et al., 2004; Murphy et al., 2005; Parker and Clasper, 2007; Sharma and Salhotra, 2012). Until now, there are few studies focusing on the NMJ-organizing molecules in tourniquet/IR skeletal muscle injuries. Although our previous studies reported that treatment with dexamethasone (a clinically used, potent anti-inflammatory drug) promotes the functional recovery of the NMJ and skeletal muscle in a mouse model of tourniquet/IR injuries (Tu et al., 2020; Zhang et al., 2017), dexamethasone cannot be used for a long time due to its severe side effects. Exploring the mechanisms responsible for tourniquet/IR injuries and finding potential therapeutic targets are crucial for improving outcomes and quality of life for limb trauma patients with tourniquet/IR complications. In the present study, therefore, we explored whether the down-expression of rapsyn is involved in the morphological damage and functional impairment of the NMJ and skeletal muscle in tourniquet/IR. Our findings could identify rapsyn as a key therapeutic target to improve NMJ and skeletal muscle function after tourniquet/IR injuries.

## Materials and methods

### Animals

We only used male mice in the present study although we understood that sex-based differences in skeletal muscle fiber-type composition, capillary density and function could affect tourniquet/IR-induced hindlimb injuries. The fact is that if male and female mice are body weight-matched, the females are older than the males; if they are age-matched, the males are heavier than the females. When we use an unadjustable rubber band to induce hindlimb tourniquet/IR, the difference of both age and body weight (i.e., limb dimension) can affect the stability and replication of the mouse model with hindlimb tourniquet/IR. For the sake of clarify and focus, therefore, only male mice were used in the present study.

Male C57BL/6 mice weighing 22–25 g at 10 weeks of age (Jackson Laboratories, Bar Harbor, Maine, United States) were housed under controlled temperature and humidity with a 12:12-h dark–light cycle and provided water and mouse chow *ad libitum*. All experimental procedures were approved by the University of Nebraska Medical Center Institutional Animal Care and Use Committee (IACUC 19-008-03-FC) and were carried out in accordance with the National Institutes of Health (NIH Publication No. 85-23, revised 1996). To achieve robust and unbiased data, all animals and tissue samples were blinded during experimentation (including the allocation) and data acquisition until statistical analysis. After *in vivo* experiments were completed, mice were euthanized with i.p. injection of 0.52 mL/kg of Fatal-Plus euthanasia (about 200 mg/kg pentobarbital, DBA Med-Vet International, Mettawa, IL, United States).

## Induction of hindlimb tourniquet/IR

Tourniquet/IR was induced as described previously (Tu et al., 2017). Briefly, under anesthesia (2% isoflurane inhalant), an orthodontic rubber band was placed at the hip joint of the left hindlimb to block the blood flow to the hindlimb. After 3 h of unilateral hindlimb ischemia, the orthodontic rubber band was released to initiate reperfusion for 6 weeks. Sham-operated mice underwent the same protocol except for no ischemia, who served as control animals. To confirm tourniquet/IR, hindlimb blood flow was measured by Laser Doppler Speckle Imaging (LDSI) System (RFLSI-ZW, RWD Life Science Inc., Dover DE). At the beginning of the 2nd week of reperfusion, 50 µL of Ad-m-RAPSN cDNA (ADV-270147, 5.2 × 10^10^ PFU/mL, VECTOR BIOLABS, Malvern, PA) was injected into the tourniquet/IR gastrocnemius muscle in two 25 µL aliquots delivered 20 min apart by Hamilton microliter syringe. 50 μL of LV-m-Rapsyn shRNA mix (TL501861V, 1 × 10^7^ TU/mL, OriGene Technologies, Rockville, MD) was injected into gastrocnemius muscle of sham mice. Ad-GFP (1,060, 1 × 10^10^ PFU/mL, VECTOR BIOLABS, Malvern, PA) or LV-shRNA scramble (TR30021V, 1 × 10^7^ TU/mL, OriGene Technologies, Rockville, MD) were served as an injection control, respectively. Terminal experiments were performed in all tourniquet/IR or sham mice as illustrated in Figure 1. Selection of 6-week tourniquet/IR as the experimental endpoint was based on our previous studies and preliminary data in which we found that there was no further recovery of NMJ and skeletal muscle function in mice with longer tourniquet/IR (8–12 weeks). The numbers of mice for each experimental measurement were provided in Supplementary Table S1.

**FIGURE 1:**
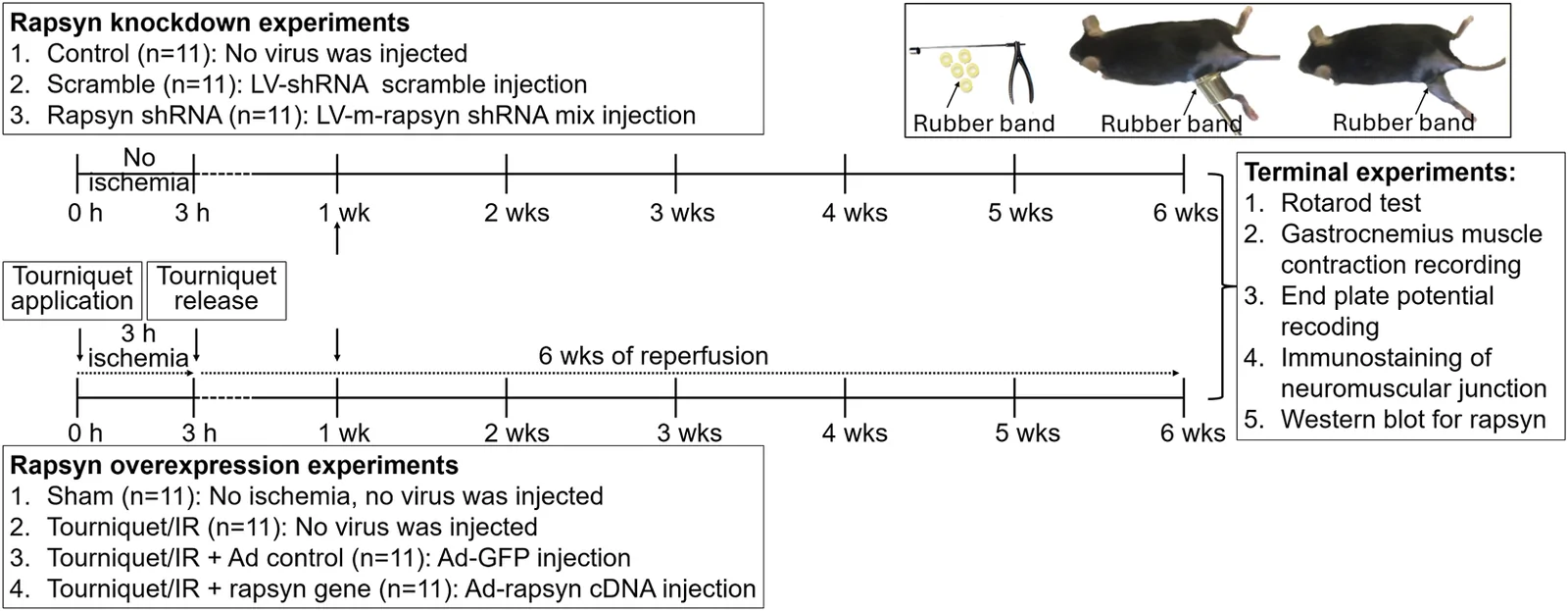
Study design, timeline, and interventions. Right top panel: an orthodontic rubber band was placed at the hip joint of the left hindlimb using a McGivney hemorrhoidal ligator to induce tourniquet/IR. Ad: Adenovirus; GFP: green fluorescent protein; LV: Lentivirus; shRNA: short hairpin RNA; tourniquet/IR: tourniquet induced ischemia/reperfusion.

### Rotarod test

Mice were placed on a rotarod (76-0772 Rota Rod, Harvard Apparatus, Holliston, MA) and subjected to 3 successive trials in 3 consecutive days. In each trial, the rotating speed gradually increased from 4 to 40 RPM in a period of 5 min The latency to fall and speed at the fall were calculated as individual performance.

### Gastrocnemius muscle contractile force

Muscle contraction was recorded as described previously (Tu et al., 2022). Briefly, under anesthesia (800 mg/kg urethane and 40 mg/kg chloralose) the distal end of the gastrocnemius muscle was connected to a force transducer (AD Instruments, Colorado Springs, CO). The distal end of the left sciatic nerve was stimulated (0.4 mA, 0.1 ms pulse, 1 s duration at 50 Hz, 100HZ, 150 Hz). The muscle contractile force was recorded and analyzed by PowerLab 8/30 Data Acquisition Systems with LabChart 7 (AD Instruments, Colorado Springs, CO).

### Electrophysiological recording of the endplate potential (EPP) *in situ*

The EPP was recorded as described previously (Tu et al., 2022). To avoid the influence of muscle contraction on the EPP recording, a specific muscle Na^+^ channel blocker, μ-conotoxin GIIIB (4 μM, 200 μL, C-270, alomone labs, Jerusalem) was locally injected into the gastrocnemius muscle. The EPPs were recorded by intracellular recording technique under the sciatic nerve stimulation (0.3 mA, 50 Hz, 0.1 ms). The amplitude of EPPs was analyzed by PowerLab 8/30 Data Acquisition System with LabChart 7 (AD Instruments, Colorado Springs, CO).

### Immunohistochemistry of the neuromuscular junction (NMJ)

Sample preparation and measurement of the NMJ morphology were conducted as described previously (Tu et al., 2017). The isolated gastrocnemius muscle was divided into small longitudinal segments for postfix. After blocking with PBS containing 0.5% Triton and 1% BSA for 1 h, the longitudinal segments were incubated with mouse anti-neurofilament 200 (N0142, Sigma-Aldrich, St. Louis, MO) and rabbit anti-synaptophysin (MA5-16402, ThermoFisher Scientific, Waltham, MA) antibodies for axon and nerve terminal labeling or mouse anti Rapsyn (MA1-746, ThermoFisher Scientific, Waltham, MA). Then the segments were incubated with Alexa Fluor® 647 labeled donkey anti-mouse (A32787, ThermoFisher Scientific, Waltham, MA), anti-rabbit (A32795, ThermoFisher Scientific, Waltham, MA) IgGs, and Alexa Fluor® 594 labeled α-Bungarotoxin (α-BTX, B13423, ThermoFisher Scientific, Waltham, MA). Finally, the muscle segment images were captured using a confocal microscope (Zeiss LSM 710).

In each muscle segment, 5 different regions were selected to obtain Z-stack images of the NMJ and to analyze the NMJ structure by ImageJ software (NIH Image, https://imagej.net/ij/) on en-face NMJs. The nAChR areas in NMJs labeled with α-BTX were used to calculate the whole area per nAChR cluster and the number of discrete fragments per nAChR cluster. A fragmented nAChR cluster was defined when the number of discrete fragments per nAChR cluster ≥5.

### Western blot

Sample preparation and Western blot analysis were conducted as described previously (Tu et al., 2021). Briefly, the gastrocnemius muscle was homogenized with a lysing buffer (10 mM Tris, 1 mM EDTA, 150 mM NaCl, 1% SDS, 1 mM PMSF; pH 7.4) plus protease inhibitor cocktail (100 μL/mL, P2714, Sigma-Aldrich, St. Louis, MO). Protein samples (30 μg/well) loaded in the stacking gel were separated on a 9%–15% sodium dodecyl sulfate (SDS)-polyacrylamide running gel and then transferred onto PVDF membrane (IPVH00010, EMD Millipore, Billerica, MA). After blocking with PBS blocking buffer (927-70001, LI-COR, Lincoin, NE) for 1 h, the membrane was incubated with rabbit anti-rapsyn (GTX64777, 1:1,000. GenTex, Irvine, CA), or mouse anti-HSC70 (sc-7298, 1:4000. Santa Cruz Biotechnology, Dallas, TX). After incubating with goat anti-rabbit IgG-IRDye 800CW (926-32211, 1:5000. LI-COR, Lincoin, NE), or conjugated goat anti-mouse IgG-IRDye 680LT (926-68020, 1:5000. LI-COR, Lincoin, NE), the membrane was scanned by Odyssey DLx (9142-02, LI-COR, Lincoin, NE) and analyzed using ImageJ software (NIH Image). HSC70 was a housekeeping protein to normalize protein intensity of rapsyn.

### Statistical analysis

All data are presented as means ± SE. SigmaPlot 12 was used for data analysis. A one-way ANOVA with *post hoc* Bonferroni test was used to determine statistical significance for multi group comparisonNormal distribution of data was confirmed with Kolmogorov-Smirov test and equal variance with Levene’s test. Statistical significance was accepted when p < 0.05.

## Results

### Tourniquet/IR results in neuromuscular junction fragmentation

Our previous studies showed that tourniquet/IR seriously damaged the gastrocnemius muscle, such as necrosis and apoptosis at the 1st day of reperfusion (Tu et al., 2021; Tran et al., 2012; Tran et al., 2011). The broken muscle fibers were still observed (Supplementary Figure S1) and dystrophin started shifting to the cytoplasm from the cell membrane (Supplementary Figure S2) in the 1st week of reperfusion. During the 2nd week of reperfusion, there were no broken muscle fibers and dystrophin was in sarcolemma (Supplementary Figures S1, S2). It suggests that the broken muscle fibers were removed and new myotubes were formed during the 2nd week of reperfusion. At this time, the virus-delivered rapsyn gene was injected into the muscle to increase rapsyn expression. The efficacy of viral transfection was about 71% ± 3.8% calculated in muscle cross section (Supplementary Figure S3), which was further validated by Western blot.

After 6 weeks of tourniquet/IR, NMJs in the gastrocnemius muscle displayed significantly fragmented nAChR clusters with low rapsyn expression (Figures 2, 3). Rapsyn colocalized with nAChRs in the gastrocnemius muscle and displayed similar fragment as nAChR clusters in the tourniquet/IR-injured muscle (Figure 2A). In the sham mice, the number of fragments in each nAChR cluster and the fragmented NMJ were 1.7% ± 0.2% and 3.8% ± 1.7% (Figures 3B,C). Compared to the sham mice, the number of fragments and fragmented NMJ per nAChR cluster significantly increased in the tourniquet/IR mice (8.5% ± 0.8% and 74.1% ± 6.1%; P < 0.05 vs. sham) (Figures 3B,C). Although the total area per nAChR cluster was not changed (403.6 ± 15.9 µm^2^ in tourniquet/IR vs. 417.2 ± 25.9 µm^2^ in sham, P > 0.05) (Figure 3D), the area per fragment in tourniquet/IR mice was reduced (88.5 ± 8.3 µm^2^ in tourniquet/IR vs. 312.7 ± 24.4 µm^2^ in sham, P < 0.05) (Figure 3E). Compared to the sham mice, tourniquet/IR significantly reduced sciatic nerve-stimulated EPPs (18.2 ± 0.9 mV in tourniquet/IR vs. 27.6 ± 0.7 mV in sham, P < 0.05) (Figures 4A,B) and muscle contraction (45.8 ± 3.5 g, 81.4 ± 5.0 g, and 86.0 ± 6.0 g in tourniquet/IR vs. 77.8 ± 3.4 g, 109.3 ± 2.0 g, and 112.4 ± 2.2 g in sham at 50 Hz, 100 Hz, and 150 Hz stimulation, respectively) (Figures 4C,D). The data from Rotarod test showed that tourniquet/IR reduced the animal ability to stay on the rotating rod with speed acceleration (106.3 ± 13.0 s for the latency to fall and 16.2 ± 1.5 rpm for the speed at fall), compared to sham mice (172.9 ± 19.3 s for the latency to fall and 24.8 ± 2.3 rpm for the speed at fall, P < 0.05) (Figures 4E,F).

**FIGURE 2:**
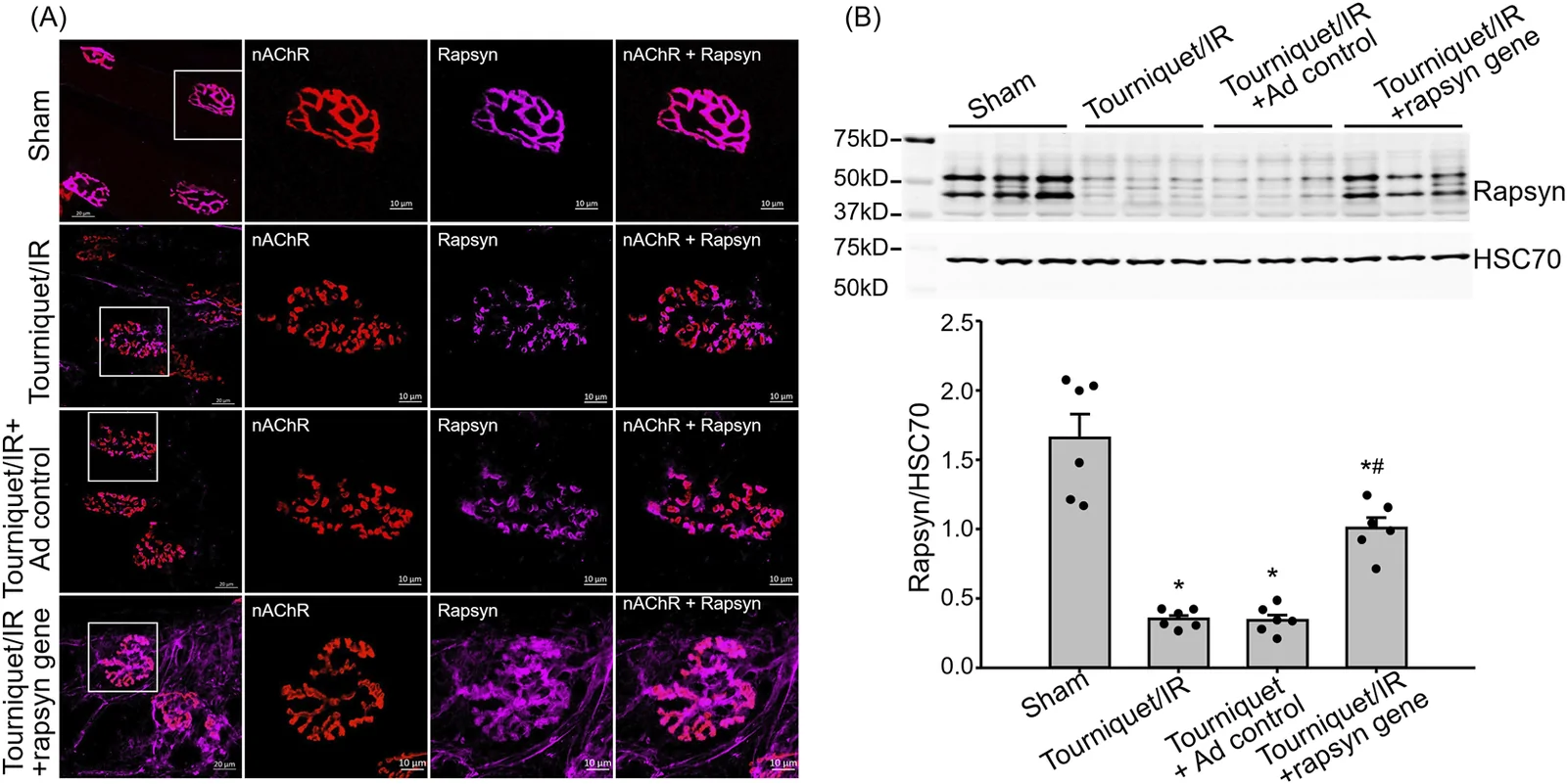
Localization (**(A)** purple color) and expression **(B)** of rapsyn in gastrocnemius muscles from sham, tourniquet/IR, tourniquet/IR + Ad control, and tourniquet/IR + rapsyn gene mice. Alpha-bungarotoxin (BTX; red color) was used to label nicotinic acetylcholine receptor (nAChR) clusters. Data are mean ± SEM, n = 6 mice in each group, *P < 0.05 vs. control; ^#^ *P* < 0.05 vs. tourniquet/IR.

**FIGURE 3:**
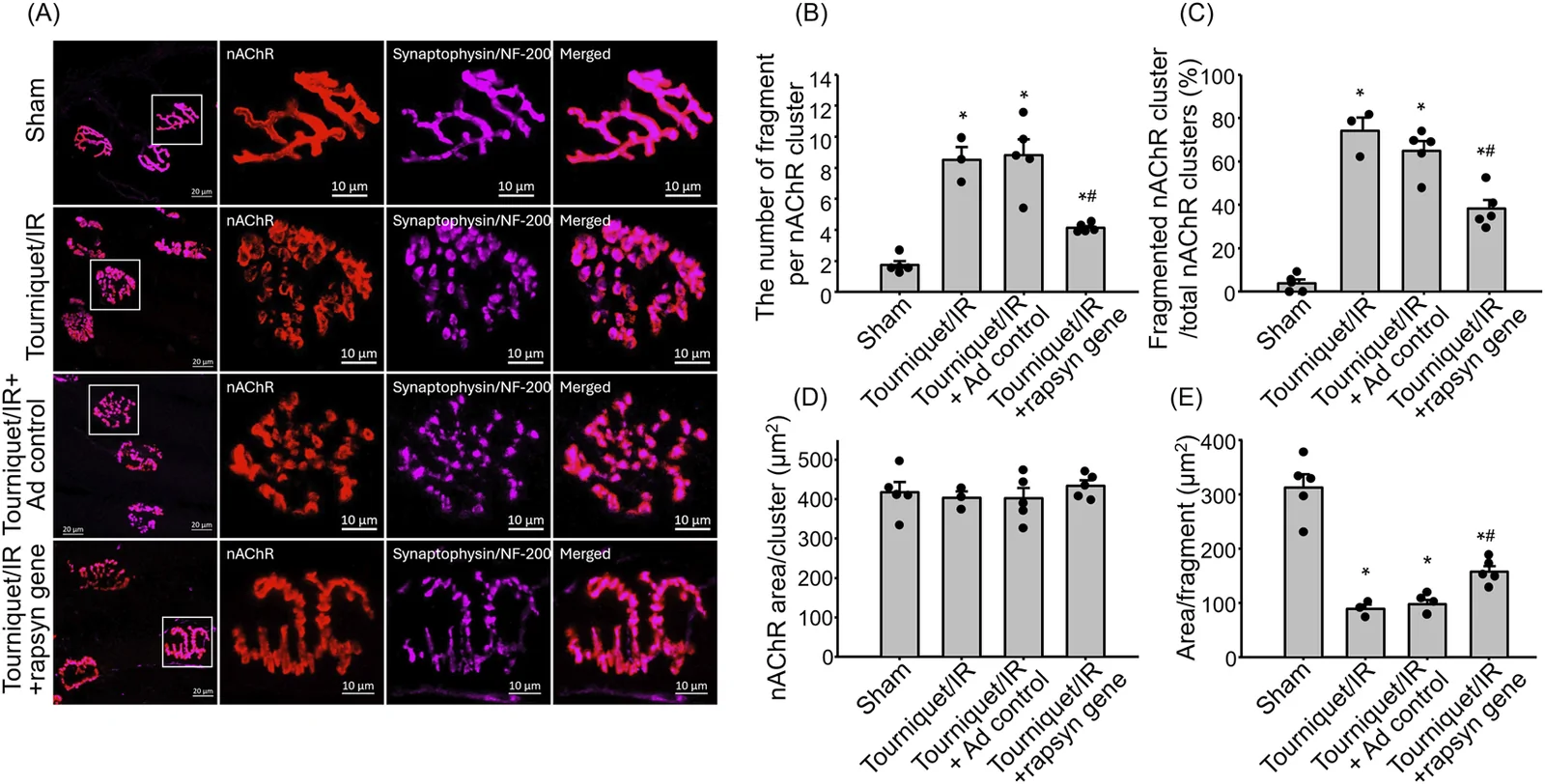
Rapsyn overexpression improved morphological recovery of the neuromuscular junction (NMJ) in the gastrocnemius muscle from tourniquet induced ischemia/reperfusion (tourniquet/IR) mice. **(A)** Representative images of NMJs in the gastrocnemius muscle from sham, tourniquet/IR, tourniquet/IR + Ad control, and tourniquet/IR + rapsyn gene mice. Synaptophysin and neurofilament-200 (purple color) and α-bungarotoxin (BTX; red color) were used to label motor nerve terminals and nAChR clusters. Mean data for **(B)** the number of fragments pe nAChR cluster, **(C)** fragmented nAChR cluster, **(D)** whole area per nAChR cluster, and **(E)** area per fragment. The number of discrete fragments per nAChR cluster ≥ 5 was considered as a fragmented nAChR cluster. Data are mean ± SEM. 113 NMJs from 5 sham mice, 95 NMJs from 3 mice with tourniquet/IR, 120 NMJs from 5 mice with tourniquet/IR + Ad control, and 110 NMJs from 5 mice with tourniquet/IR + rapsyn gene. *P < 0.05 vs. sham; ^#^P < 0.05 vs. tourniquet/IR.

**FIGURE 4:**
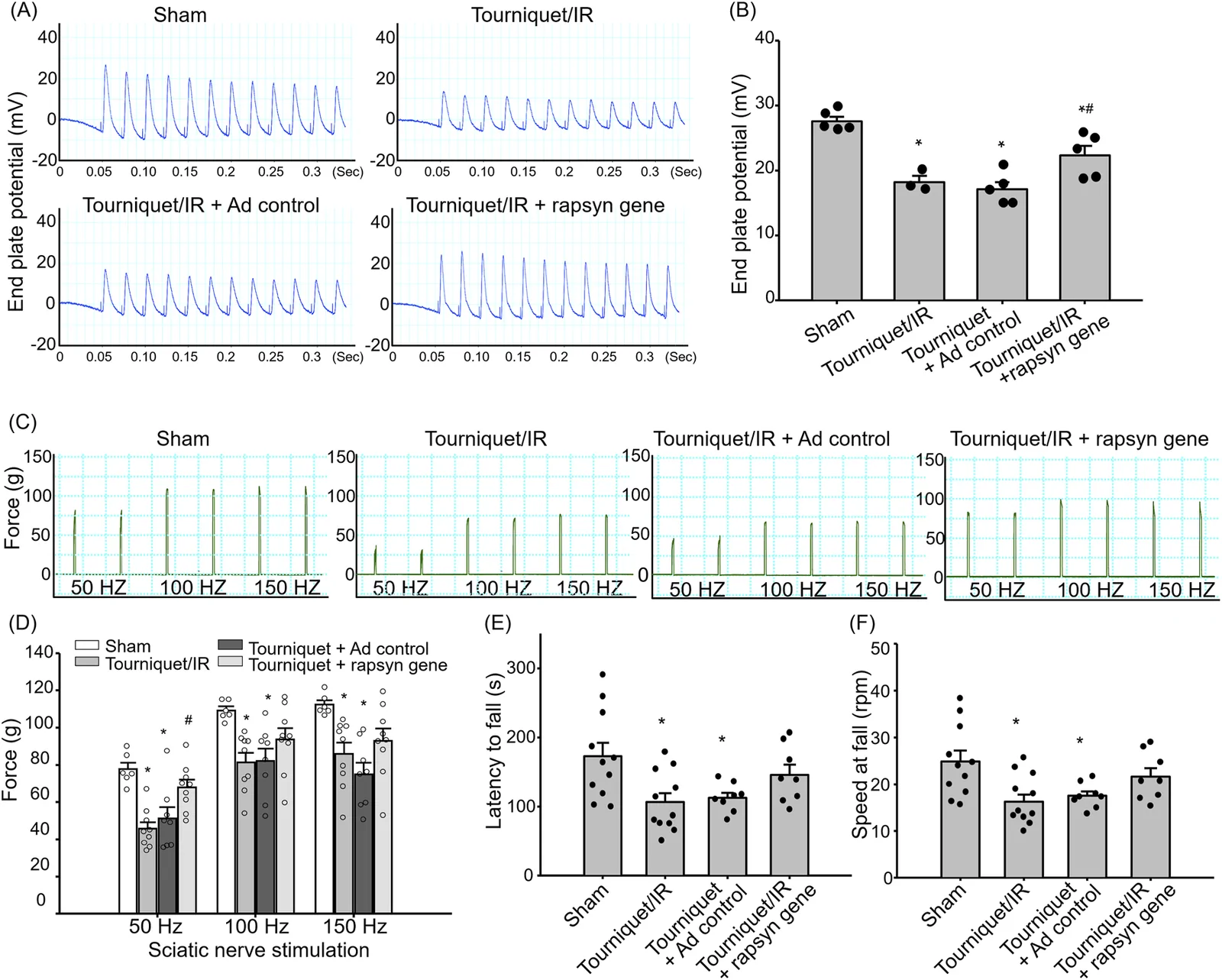
Rapsyn overexpression improved functional recovery of skeletal muscle from tourniquet induced ischemia/reperfusion (tourniquet/IR) mice. **(A)** Representative recording and **(B)** mean data for sciatic nerve-stimulated endplate potentials (EPPs) in gastrocnemius muscle from sham, tourniquet/IR, tourniquet/IR + Ad control, and tourniquet/IR + rapsyn gene mice. Data are mean ± SEM, n = 5, 3, 5, and 5 mice in sham, tourniquet/IR, tourniquet/IR + Ad control, and tourniquet/IR + rapsyn gene groups, respectively. *P < 0.05 vs. sham; ^#^ *P* < 0.05 vs. tourniquet/IR. **(C)** Representative *in situ* recording and **(D)** mean data of gastrocnemius muscle tetanic contraction elicited by sciatic never stimulation at 50, 100, and 150 Hz in sham, tourniquet/IR, tourniquet/IR + Ad control, and tourniquet/IR + rapsyn gene mice. Data are mean ± SEM, n = 6, 9, 8, and 9 mice in sham, tourniquet/IR, tourniquet/IR + Ad control, and tourniquet/IR + rapsyn gene groups, respectively. *P < 0.05 vs. sham; **(E)** Average latency to fall and **(F)** speed at fall in accelerating rotarod test. Data are mean ± SEM, n = 11, 11, 8, and 8 mice in sham, tourniquet/IR, tourniquet/IR + Ad control, and tourniquet/IR + rapsyn gene groups, respectively. *P < 0.05 vs. sham; ^#^ *P* < 0.05 vs. tourniquet/IR.

### Rapsyn overexpression improves recovery of the NMJ in the injured muscle

The Local transfection of an Ad-m-RAPSN gene into the tourniquet/IR-injured gastrocnemius muscle increased rapsyn expression and mitigated the morphological damage of nAChR clusters, compared to tourniquet/IR alone (Figures 2, 3). The rapsyn level increased 2.9-fold in tourniquet/IR with Ad-rapsyn gene transfection, compared to tourniquet/IR with Ad-control transfection (Figure 2). The number of fragments per nAChR cluster and fragmented NMJs were reduced in tourniquet/IR + rapsyn gene mice (4.1% ± 0.1% and 38.2% ± 4.0%, P < 0.05 vs. tourniquet/IR) (Figures 3B,C). The whole area per nAChR cluster was not significantly changed in tourniquet/IR + rapsyn gene mice (433.9 ± 13.7 µm^2^, P > 0.05 vs. tourniquet/IR) (Figure 3D), while the area per fragment was increased in tourniquet/IR + rapsyn gene mice (157.6 ± 10.2 µm^2^, P < 0.05 vs. tourniquet/IR) (Figure 3E). The motor nerve innervation and terminal occupancy in the NMJ were not different among groups (Supplementary Figure S4). Additionally, treatment with the Ad-m-RAPSN gene also improved NMJ function in the tourniquet/IR-injured muscle, demonstrated by increase in the sciatic nerve-stimulated EPPs (22.3 ± 1.5 mV, P < 0.05 vs. tourniquet/IR alone) (Figures 4A,B). Although rapsyn overexpression mitigated fragmentation of nAChR clusters and improved NMJ function, structure and function of the damaged NMJs were not normalized to the levels in sham mice (P < 0.05 vs. sham) (Figures 3B,C,E, 4B). More interestingly, overexpressed rapsyn in the injured muscle increased sciatic nerve-stimulated skeletal muscle contraction at 50 Hz stimulation (67.9 ± 4.1 g, P < 0.05 vs. tourniquet/IR) but not 100 Hz (93.8 ± 5.9 g, P > 0.05 vs. tourniquet/IR) and 150 Hz stimulation (93.0 ± 6.5 g, P > 0.05 vs. tourniquet/IR) (Figures 4C,D). In the rotarod test, muscle contractile function showed an improved trajectory with Ad-m-RAPSN treatment (146.0 ± 14.5 s for the latency to fall and 21.6 ± 1.8 rpm for the speed at fall), but there was no statistical significance compared to tourniquet/IR alone (P > 0.05, Figures 4E,F).

### Rapsyn knockdown impairs neuromuscular function

To further clarify the role of rapsyn in morphology and function of the NMJ, the lentivirus-delivered rapsyn shRNA was used to knockdown rapsyn expression in normal gastrocnemius muscles. The local injection of the LV-m-rapsyn shRNA significantly decreased rapsyn expression in the gastrocnemius muscle (Figure 5A) After rapsyn knockdown, the area per nAChR cluster was not significantly reduced (368.6 ± 14.9 µm^2^ in rapsyn shRNA vs. 417.9 ± 23.9 µm^2^ in control, P > 0.05) (Figure 5C), while rapsyn knockdown induced significant increase in the number of fragments per nAChR cluster and fragmented NMJs (4.6% ± 0.4% and 41.9% ± 5.8% in rapsyn shRNA vs. 1.8% ± 0.1% and 2.4% ± 1.0% in control, P < 0.05) (Figures 5B,D,E) and marked decrease in the area per fragment (142.9 ± 20.6 µm^2^ in rapsyn shRNA vs. 306.1 ± 21.0 µm^2^ in control, P < 0.05) (Figure 5F). However, rapsyn knockdown did not alter motor nerve innervation and terminal occupancy in the NMJ (Supplementary Figure S5). For the functional measurement, rapsyn knockdown reduced the amplitude of the sciatic nerve-stimulated EPPs (22.3 ± 0.6 mV in rapsyn shRNA vs. 26.9 ± 0.7 mV in control, P < 0.05) (Figures 6A,B). Additionally, rapsyn knockdown also attenuated muscle contractile function, measured by sciatic nerve stimulated muscle contraction and rotarod test, compared to the control group (Figures 6C–F). However, scrambled shRNA did not affect muscle contractile function (Figures 6C–F). Interestingly, rapsyn knockdown had less effect on the muscle contraction induced by 50 Hz sciatic nerve stimulation (65.8 ± 7.5 g in rapsyn shRNA vs. 73.3 ± 3.0 g in control, P > 0.05), while it significantly impaired the muscle contraction induced by high frequency sciatic nerve stimulation (Figure 6D).

**FIGURE 5:**
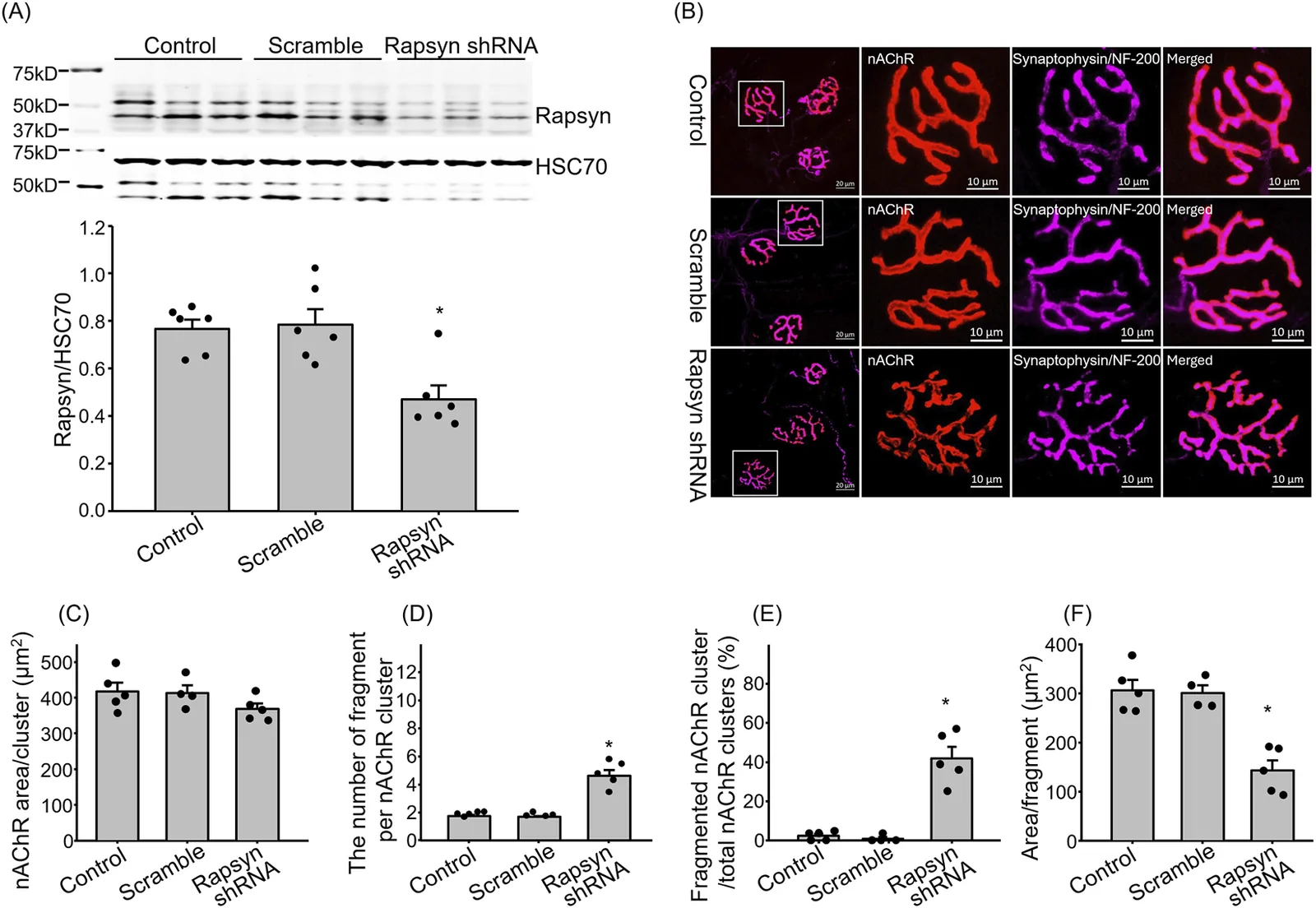
LV-m-rapsyn shRNA injection decreased rapsyn expression and promoted neuromuscular junction fragmentation in gastrocnemius muscles in control mice. **(A)** Rapsyn expression in the gastrocnemius muscle in control mice (control), mice treated with LV-scramble (scramble) or LV-m-rapsyn shRNA (rapsyn shRNA). Data are mean ± SEM, n = 6 mice in each group, *P < 0.05 vs. control. **(B)** Representative images of NMJ, including nerve terminals (Synaptophysin + NF-200) and nAChRs, located in gastrocnemius muscles from control mice (control), mice treated with LV-scrambled shRNA (scramble) or LV-m-rapsyn shRNA (rapsyn shRNA). Synaptophysin and neurofilament-200 (purple color) and α-bungarotoxin (BTX; red) were used to label motor nerve terminals and nAChR clusters. Mean data for **(C)** whole area per nAChR cluster, **(D)** the number of fragments pe nAChR cluster, **(E)** fragmented nAChR cluster and **(F)** area per fragment. The number of discrete fragments per nAChR cluster ≥ 5 was considered as a fragmented nAChR cluster. Data are mean ± SEM. 130 NMJs from 5 control mice, 109 NMJs from 4 mice with scramble shRNA, and 206 NMJs from 5 mice with rapsyn shRNA. *P < 0.05 vs. control.

**FIGURE 6:**
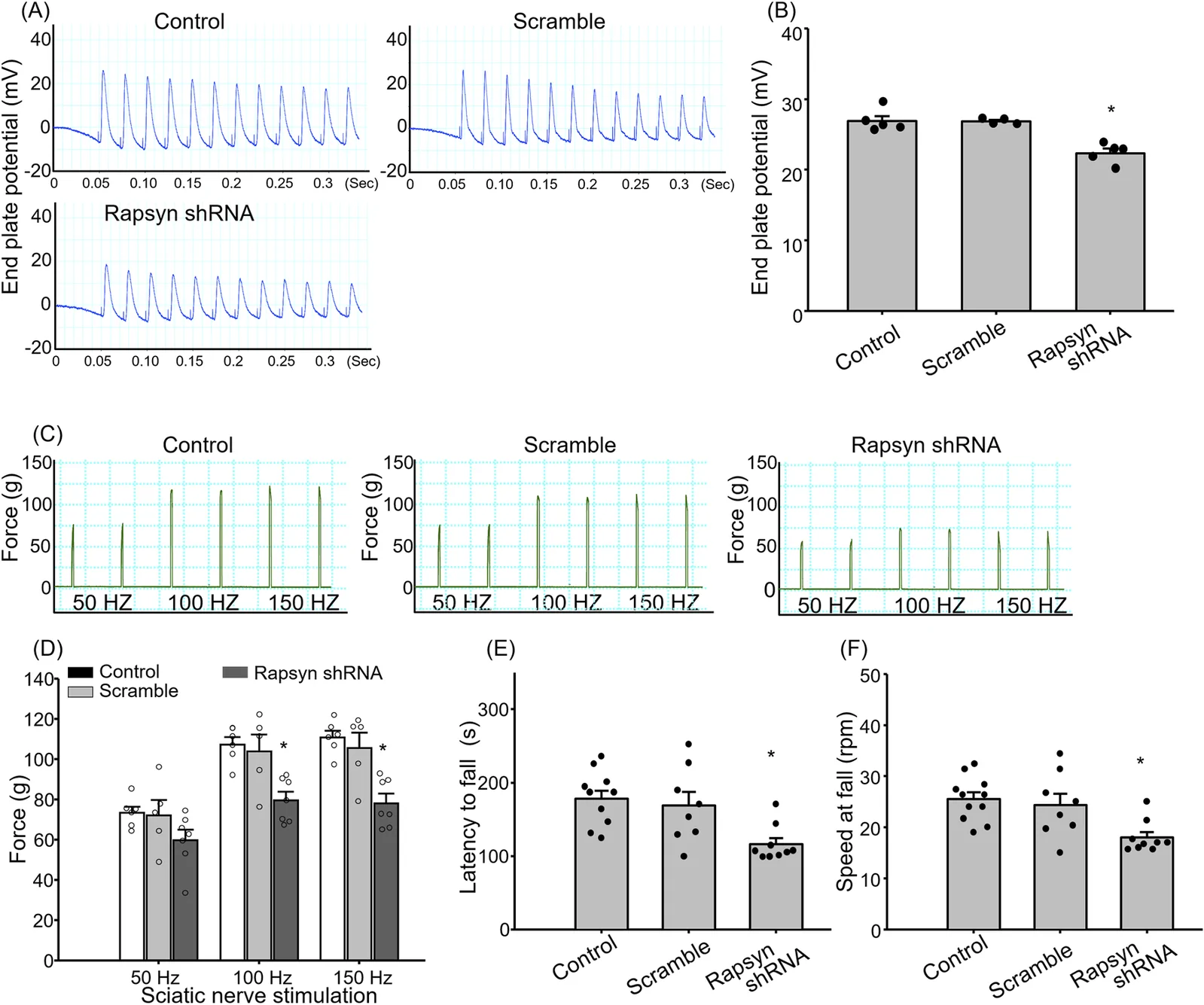
LV-m-Rapsyn shRNA injection impaired skeletal muscle function in control mice. **(A)** Representative recording and **(B)** mean data for sciatic nerve-stimulated endplate potentials (EPPs) in the gastrocnemius muscle from control mice (control), mice treated with LV-scrambled shRNA (scramble), and LV-m-Rapsyn shRNA (rapsyn shRNA). Data are mean ± SEM, n = 5, 4, and 5 mice in control, scramble, and rapsyn shRNA groups, respectively. *P < 0.05 vs. control. **(C)** Representative *in situ* recording and **(D)** mean data of gastrocnemius muscle tetanic contraction elicited by sciatic never stimulation at 50, 100, and 150 Hz in control mice (control), mice treated with scramble, or rapsyn shRNA. Data are mean ± SEM, n = 6, 5, 7 in control, scramble, and rapsyn shRNA groups, respectively. *P < 0.05 vs. control. **(E)** Average latency to fall and **(F)** speed at fall in accelerating rotarod test, n = 11, 8, and 9 mice in control, scramble, and rapsyn shRNA groups, respectively. Data are mean ± SEM, *P < 0.05 vs. control.

## Discussion

In the present study, we found that the protein level of rapsyn was reduced in the gastrocnemius muscle after tourniquet/IR. Tourniquet/IR also induced the fragmentation of the nAChR clusters and reduction of the EPP amplitudes and muscle contraction. Treatment with the Ad-m-RAPSN gene in the injured gastrocnemius muscle increased rapsyn expression and promoted the recovery of NMJ structure and function as well as skeletal muscle contraction. Conversely, treatment with the LV-m-rapsyn shRNA in the normal gastrocnemius muscle could mimic tourniquet/IR to decrease rapsyn expression and further resulted in the fragmentation of the nAChR clusters and impairment of the NMJs and skeletal muscles. These results suggest that the upregulation of rapsyn levels could be a therapeutic strategy to protect the hindlimb against tourniquet/IR-related fragmentation of nAChR clusters and dysfunction of the NMJs and skeletal muscles.

Rapsyn plays a crucial role in the nAChR clustering at the postsynaptic membrane and NMJ maintenance in the skeletal muscle cells (Xing et al., 2020). Rapsyn clusters the nAChRs (Gautam et al., 1995) and increases the nAChR half-life on the cell membrane (Phillips et al., 1997). In rapsyn-deficient mice, the synaptic activity is decreased due to the lower density of nAChRs in the postsynaptic membrane, although motor axons can form specialized presynaptic nerve terminals and the nAChRs are also concentrated at synaptic sites (Gautam et al., 1995). Rapsyn overexpression stabilizes the nAChRs in the motor endplates (Martinez-Martinez et al., 2007) with the trafficking of the nAChRs from the cell cytosol to the outer membrane (Han et al., 2000) and prevents nAChR loss in an animal model of autoimmune myasthenia gravis (Losen et al., 2005). Our present study confirmed that rapsyn levels in the skeletal muscle could affect the NMJ morphology and function. Tourniquet/IR decreased the levels of rapsyn protein accompanied by fragmented nAChR clusters and reduced EPPs. Overexpression of the rapsyn protein in the tourniquet/IR-injured skeletal muscle mitigated tourniquet/IR-induced fragmentation of the nAChR clusters and NMJ dysfunction. Conversely, downregulation of the rapsyn protein in the normal skeletal muscle mimicked tourniquet/IR to result in the fragmentation of the nAChR clusters and impair NMJ and skeletal muscle function. These results further confirm that the low level of the rapsyn protein is involved in the impairment of the nAChR clusters and NMJ dysfunction in tourniquet/IR-injured skeletal muscles. Additionally, our present study found that rapsyn overexpression rescued tourniquet/IR-injured skeletal muscle contraction induced by only low frequency (50 Hz) but not high frequency (100 and 150 Hz) nerve stimulation, whereas knockdown of the rapsyn protein in the normal skeletal muscle significantly impaired the muscle contraction induced by high frequency (100 and 150 Hz) but not low frequency (50 Hz) nerve stimulation. From these results, we assume that an intact NMJ structure is more crucial for the strong muscle contraction triggered by high frequency nerve stimulation.

In our current study, targeted intervention with rapsyn overexpression partially restored the NMJ structure and function in tourniquet/IR-injured skeletal muscles. These findings highlight the multifactorial nature of NMJ maintenance and repair. NMJ normality requires extensive communication and integration among the three components of the tripartite synapse: presynaptic motoneurons with terminals, postsynaptic muscle fibers (including nAChRs), and Schwann cells. Even though the intracellular mechanisms for the nAChR clustering are not totally clear, there are several signaling complexes to regulate the nAChR transport, assembling and stabilization, such as Lrp4, Dok7, MuSK, dystrophin, α-actinin, etc., (Li et al., 2018). These signaling pathways could also be potential factors to contribute to tourniquet/IR-induced nAChR fragmentation and NMJ dysfunction. Our previous study displayed that dystrophin almost disappeared from sarcolemma after tourniquet application (Tu et al., 2021). Therefore, full restoration of the NMJ architecture and function after tourniquet/IR injuries likely depends on the coordination of numerous signaling pathways and cellular partners. When only rapsyn overexpression was treated in tourniquet/IR injured skeletal muscles in the present study, we believe that the above compensatory mechanisms and multifactorial coordination remain insufficiently engaged in complete recovery of damaged NMJs.

Although rapsyn overexpression improved the NMJ transmission and sciatic nerve-stimulated gastrocnemius muscle contraction in tourniquet/IR mice, it only brought a tendentious, nonsignificant improvement in the skeletal muscle contractile function, measured by a rotarod test in conscious animals. We clearly recognize that rotarod test is usually used to evaluate motor and skeletal muscle coordination (Deacon, 2013; Shiotsuki et al., 2010; Graber et al., 2021; Trovato et al., 2022), which includes all skeletal muscles involved in motor activity. Therefore, one possible explanation for the result discrepancy measured by different techniques in the present study is that rapsyn overexpression was limited in the gastrocnemius muscle but not tourniquet/IR-injured unilateral hindlimb muscles.

Although our current study confirmed that the rapsyn downregulation was involved in tourniquet/IR-impaired NMJ structure and function, the mechanisms underlying the rapsyn downregulation are unclear. Previous studies reported that Wnt3a-activated β-catenin reduced the rapsyn expression and further inhibited the nAChR clustering (Henriquez and Salinas, 2012; Stamatakou and Salinas, 2014; Wang and Luo, 2008; Wang et al., 2008). Our pilot data also found that tourniquet/IR increased the protein expression of β-catenin in the skeletal muscle (Tu et al., 2025). Therefore, it is possible that the β-catenin overexpression negatively regulates rapsyn transcription to reduce the protein expression of rapsyn in the tourniquet/IR mice. Additionally, another possibility is that rapsyn degradation via ubiquitination of rapsyn protein contributes to the rapsyn downregulation in the tourniquet/IR mice. Further study is needed to clarify these mechanisms.

A limitation of this study is that Ad-m-RAPSN transfection was not exclusively restricted to skeletal muscle fibers. Normally, rapsyn expresses in the skeletal muscle and is co-localized with nAChRs on the skeletal muscle membrane. However, it was reported that rapsyn has little expression in the brain and induces lysosome clustering (Liao et al., 2023). So far, the role of rapsyn in the peripheral nerve is not reported. Although exogenous Ad-m-RAPSN transfection might induce rapsyn expression in motor axons (Figure 2A), it affects neither motor nerve innervation nor terminal occupancy in the NMJ (Supplementary Figure S4).

As shown in Figure 7, the rapsyn downregulation was involved in the fragmentation of the nAChR clusters and the NMJ and skeletal muscle dysfunction in the tourniquet/IR mice. The exogenous overexpression of rapsyn via *in vivo* Ad-m-RAPSN injection into the injured skeletal muscle improved the NMJ structure and function as well as skeletal muscle contractile function in the tourniquet/IR mice. Considering the rapsyn overexpression in the injured skeletal muscle at the beginning of the 2^nd^ week following tourniquet/IR in the present study, we believe that the rapsyn upregulation after tourniquet/IR will serve as a potential therapeutic strategy for the long-term recovery of NMJ and skeletal muscle function after tourniquet/IR injuries, which has clinical relevance with translational potential.

**FIGURE 7:**
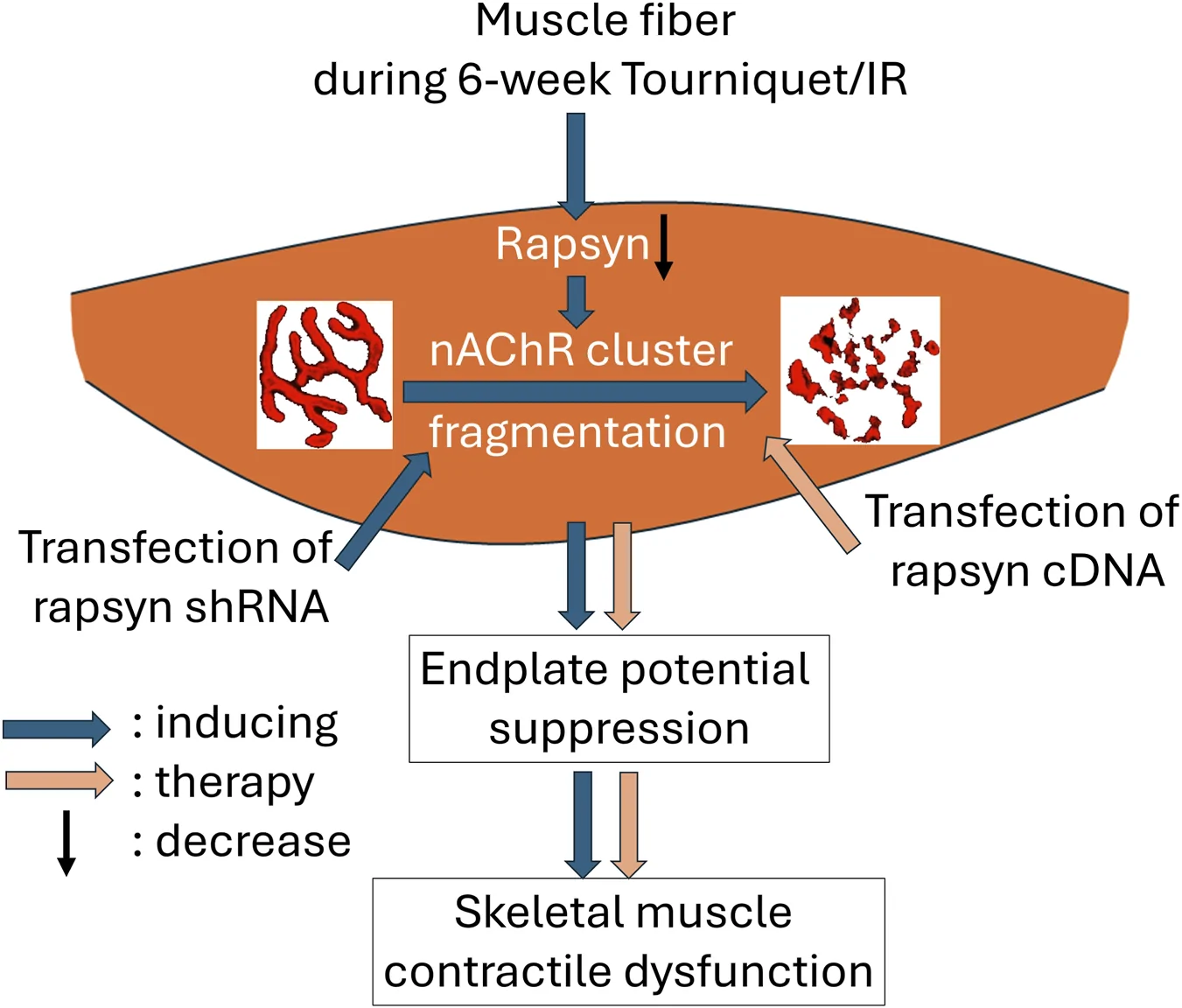
Schematic diagram illustrating the main findings of the present study. The tourniquet/IR-decreased rapsyn protein results in the nAChR cluster fragmentation and impairs the neuromuscular transmission and skeletal muscle contraction. Overexpression of the rapsyn protein mitigates the NMJ and skeletal muscle dysfunction induced by tourniquet/IR.

## Data Availability

The original contributions presented in the study are included in the article/Supplementary Material, further inquiries can be directed to the corresponding author.
