# Supplementary material for: Rapsyn overexpression improves neuromuscular junction and skeletal muscle function after tourniquet-induced mouse hindlimb ischemia-reperfusion injuries
Source: Front Bioeng Biotechnol. 2026 Oct 15;14:1906715. doi: 10.3389/fbioe.2026.1906715 (PMC13716861; doi:10.3389/fbioe.2026.1906715)
Supplement: Supplementary file 1 [file Supplementaryfile1.docx]

**Rapsyn overexpression improves neuromuscular junction and skeletal muscle function after tourniquet-induced mouse hindlimb ischemia-reperfusion injuries**

Huiyin Tu, Emmanuel Adu-Agyekum, Aaron N. Barksdale, Michael C. Wadman, Yu-Long Li*

Department of Emergency Medicine, University of Nebraska Medical Center, Omaha, NE 68198, USA

*Corresponding author:

Yu-Long LI, M.D., Ph.D.

Department of Emergency Medicine

University of Nebraska Medical Center

985850 Nebraska Medical Center

Omaha, NE 68198-5850, USA

Tel.: +1-402-559-3016; Fax: +1-402-559-9659

E-mail: yulongli@unmc.edu

**Materials and Methods for Supplemental figures**

**Histological evaluation of skeletal muscle damage**

Gastrocnemius muscles from control, 1 week of tourniquet/IR, 2 weeks of tourniquet/IR, and 6 weeks of tourniquet/IR mice were successively fixed in Methacarn solution (300 ml methanol, 150 ml chloroform, and 50 ml acetic acid) for 48 hours, and 60% ethanol for 72 hours. The fixed muscles were processed for section and H&E staining. Stained sections were captured by a Zeiss Observer Z1 microscope for bright field (Carl Zeiss AG, Oberkochen, Germany) with digital camera (Axiocam 503).

In the other experiment, gastrocnemius muscles from control, 1 week of tourniquet/IR, 2 weeks of tourniquet/IR, and 6 weeks of tourniquet/IR mice were postfixed with 4% paraformaldehyde for 12 hours, and then soaked in 30% sucrose for 12 hours at 4°C for cryostat procedure. The muscle was cut into 20-µm-thick cross-sections in a freezing cryostat at -20°C. Tissue sections were processed with standard immunocytochemical staining procedure. Briefly, tissue sections were permeabilized with 0.3% Triton X-100 (Thermo Fisher Scientific, Waltham, MA) in PBS at room temperature for 20 minutes. After tissue sections were successively incubated with 10% normal goat serum (Jackson Immunoresearch Labs Inc.), either rabbit anti-laminin antibody (L9393, Sigma-Aldrich, St. Louis, MO) or anti-dystrophin antibody (ab15277, abcam, Cambridge, MA), and goat anti-rabbit IgG labelled with Alexa Fluor 594 (Thermo Fisher Scientific, Waltham, MA) with DAPI (D9542, MilliporeSigma, Rockville, MD), tissue immunofluorescent images were obtained using a Zeiss Observer Z1 microscope (Carl Zeiss AG, Oberkochen, Germany) with digital camera (Axiocam 105).

**Suppl Table.** Experimental flow of the numbers of mice used in each measurement.

Each group has 11 mice, and all mice were subjected to rotarod test. Because some mice couldn’t stand on the rotarod bar during the experiments, those mice were excluded from data analysis of rotarod test. After rotarod test, some mice were used to measure the muscle contraction and run western blot. Other mice were used to record the EPPs after the muscle contraction measurements. Mice subjected to EPP recording were used for immunofluorescent image analyses.

**Suppl figure 1.** Hematoxylin and eosin staining of longitudinal gastrocnemius muscle section from sham, 1 week (tourniquet/IR1wk), 2 weeks (tourniquet/IR2wk) and 6 weeks (tourniquet/IR6wk) of tourniquet induced ischemia/reperfusion mice.

**Suppl figure 2**. Immunofluorescence images of adjacent cross gastrocnemius muscle sections with laminin and dystrophin staining from sham, 1 week (tourniquet/IR1wk), 2 weeks (tourniquet/IR2wk) and 6 weeks (tourniquet/IR6wk) of tourniquet induced ischemia/reperfusion mice.

**Suppl figure 3.** Light and fluorescence images of cross gastrocnemius muscle section from tourniquet-induced ischemia/reperfusion mice**.** The muscle was injected with saline, adenoviral GFP (Ad-GTP), or lentiviral-m-rapsyn shRNA with GFP (LV-rapsyn shRNA-GFP). Total muscle fibers and GFP positive fibers were calculated in a cross section, and then the percent of GFP positive fiber in total muscle fibers was defined as transfection efficacy in this section. Nine sections from 3 mice were calculated.

**Suppl figure 4.** The effects of rapsyn overexpression on motor nerve terminals in the gastrocnemius muscle from tourniquet induced ischemia/reperfusion (tourniquet/IR) mice. Quantitative data for motor nerve terminal occupancy (%) and motor nerve innervation to nAChR clusters (%) were calculated from original NMJ images in all groups, same as Figure 3. Data are mean ± SEM, n = 5, 3, 5, and 5 mice in sham, tourniquet/IR, tourniquet/IR + Ad control, and tourniquet/IR + rapsyn gene groups, respectively. *P < 0.05 *vs*. sham; ^#^P < 0.05 *vs*. tourniquet/IR.

**Suppl figure 5.** The effects of rapsyn knockdown on motor nerve terminals in the gastrocnemius muscle from control mice. Quantitative data for motor nerve terminal occupancy (%) and motor nerve innervation to nAChR clusters (%) were calculated from original NMJ images in all groups, same as Figure 5. Data are mean ± SEM, n = 5, 4, and 5 mice in control, scramble, and rapsyn shRNA groups, respectively. **P* < 0.05 *vs.* control.

**Original gel imaging**
